# Site-specific glycan analysis of the SARS-CoV-2 spike

**DOI:** 10.1126/science.abb9983

**Published:** 2020-05-04

**Authors:** Yasunori Watanabe, Joel D. Allen, Daniel Wrapp, Jason S. McLellan, Max Crispin

**Affiliations:** 1 School of Biological Sciences, University of Southampton, Southampton SO17 1BJ, UK.; 2 Oxford Glycobiology Institute, Department of Biochemistry, University of Oxford, South Parks Road, Oxford OX1 3QU, UK.; 3 Division of Structural Biology, University of Oxford, Wellcome Centre for Human Genetics, Oxford OX3 7BN, UK.; 4 Department of Molecular Biosciences, The University of Texas at Austin, Austin, TX 78712, USA.

## Abstract

The emergence of the betacoronavirus, severe acute respiratory syndrome coronavirus 2 (SARS-CoV-2), the causative agent of coronavirus disease 2019 (COVID-19), represents a considerable threat to global human health. Vaccine development is focused on the principal target of the humoral immune response, the spike (S) glycoprotein, which mediates cell entry and membrane fusion. The SARS-CoV-2 S gene encodes 22 N-linked glycan sequons per protomer, which likely play a role in protein folding and immune evasion. Here, using a site-specific mass spectrometric approach, we reveal the glycan structures on a recombinant SARS-CoV-2 S immunogen. This analysis enables mapping of the glycan-processing states across the trimeric viral spike. We show how SARS-CoV-2 S glycans differ from typical host glycan processing, which may have implications in viral pathobiology and vaccine design.

## SARS-CoV-2 spike protein, elaborated

Vaccine development for severe acute respiratory syndrome coronavirus 2 (SARS-CoV-2) is focused on the trimeric spike protein that initiates infection. Each protomer in the trimeric spike has 22 glycosylation sites. How these sites are glycosylated may affect which cells the virus can infect and could shield some epitopes from antibody neutralization. Watanabe *et al.* expressed and purified recombinant glycosylated spike trimers, proteolysed them to yield glycopeptides containing a single glycan, and determined the composition of the glycan sites by mass spectrometry. The analysis provides a benchmark that can be used to measure antigen quality as vaccines and antibody tests are developed.

*Science* this issue p. 330

## Teaser

A mass spectrometry analysis reveals the glycan composition at all glycosylation sites on the SARS-CoV-2 spike protein.

Severe acute respiratory syndrome coronavirus 2 (SARS-CoV-2), the causative pathogen of coronavirus 2019 (COVID-19) (*1*, *2*), induces fever, severe respiratory illness, and pneumonia. SARS-CoV-2 uses an extensively glycosylated spike (S) protein that protrudes from the viral surface to bind to angiotensin-converting enzyme 2 (ACE2) to mediate host-cell entry (*3*). The S protein is a trimeric class I fusion protein, composed of two functional subunits, responsible for receptor binding (S1 subunit) and membrane fusion (S2 subunit) (*4*, *5*). The surface of the envelope spike is dominated by host-derived glycans, with each trimer displaying 66 N-linked glycosylation sites. The S protein is a key target in vaccine design efforts (*6*), and understanding the glycosylation of recombinant viral spikes can reveal fundamental features of viral biology and guide vaccine design strategies (*7*, *8*).

Viral glycosylation has wide-ranging roles in viral pathobiology, including mediating protein folding and stability and shaping viral tropism (*9*). Glycosylation sites are under selective pressure as they facilitate immune evasion by shielding specific epitopes from antibody neutralization. However, we note the low mutation rate of SARS-CoV-2 and that as yet, there have been no observed mutations to N-linked glycosylation sites (*10*). Surfaces with an unusually high density of glycans can also enable immune recognition (*9*, *11*, *12*). The role of glycosylation in camouflaging immunogenic protein epitopes has been studied for other coronaviruses (*10*, *13*, *14*). Coronaviruses form virions by budding into the lumen of endoplasmic reticulum–Golgi intermediate compartments (*15*, *16*). However, observations of complex-type glycans on virally derived material suggests that the viral glycoproteins are subjected to Golgi-resident processing enzymes (*13*, *17*).

High viral glycan density and local protein architecture can sterically impair the glycan maturation pathway. Impaired glycan maturation resulting in the presence of oligomannose-type glycans can be a sensitive reporter of native-like protein architecture (*8*), and site-specific glycan analysis can be used to compare different immunogens and monitor manufacturing processes (*18*). Additionally, glycosylation can influence the trafficking of recombinant immunogen to germinal centers (*19*).

**Fig. 1:**
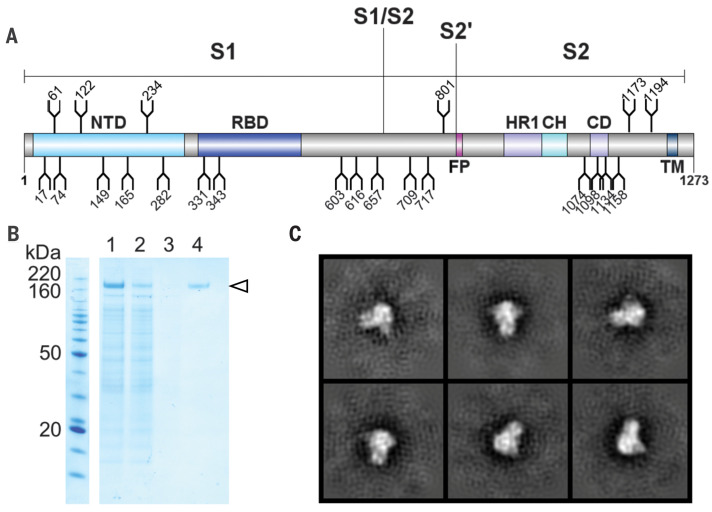
Expression and validation of the SARS-CoV-2 S glycoprotein. (**A**) Schematic representation of the SARS-CoV-2 S glycoprotein. The positions of N-linked glycosylation sequons (N-X-S/T, where X ≠ P) are shown as branches (N, Asn; X, any residue; S, Ser; T, Thr; P, Pro). Protein domains are illustrated: N-terminal domain (NTD), receptor binding domain (RBD), fusion peptide (FP), heptad repeat 1 (HR1), central helix (CH), connector domain (CD), and transmembrane domain (TM). (**B**) SDS–polyacrylamide gel electrophoresis analysis of the SARS-CoV-2 S protein (indicated by the arrowhead) expressed in human embryonic kidney (HEK) 293F cells. Lane 1: filtered supernatant from transfected cells; lane 2: flow-through from StrepTactin resin; lane 3: wash from StrepTactin resin; lane 4: elution from StrepTactin resin. (**C**) Negative-stain EM 2D class averages of the SARS-CoV-2 S protein. 2D class averages of the SARS-CoV-2 S protein are shown, confirming that the protein adopts the trimeric prefusion conformation matching the material used to determine the structure (*4*).

To resolve the site-specific glycosylation of the SARS-CoV-2 S protein and visualize the distribution of glycoforms across the protein surface, we expressed and purified three biological replicates of recombinant soluble material in an identical manner to that which was used to obtain the high-resolution cryo–electron microscopy (cryo-EM) structure, albeit without a glycan-processing blockade using kifunensine (*4*). This variant of the S protein contains all 22 glycans on the SARS-CoV-2 S protein (Fig. 1A). Stabilization of the trimeric prefusion structure was achieved by using the 2P stabilizing mutations (*20*) at residues 986 and 987, a GSAS (Gly-Ser-Ala-Ser) substitution at the furin cleavage site (residues 682 to 685), and a C-terminal trimerization motif. This helps to maintain quaternary architecture during glycan processing. Before analysis, supernatant containing the recombinant SARS-CoV-2 S was purified by size exclusion chromatography to ensure that only native-like trimeric protein was analyzed (Fig. 1B and fig. S1). The trimeric conformation of the purified material was validated by using negative-stain EM (Fig. 1C).

**Fig. 2:**
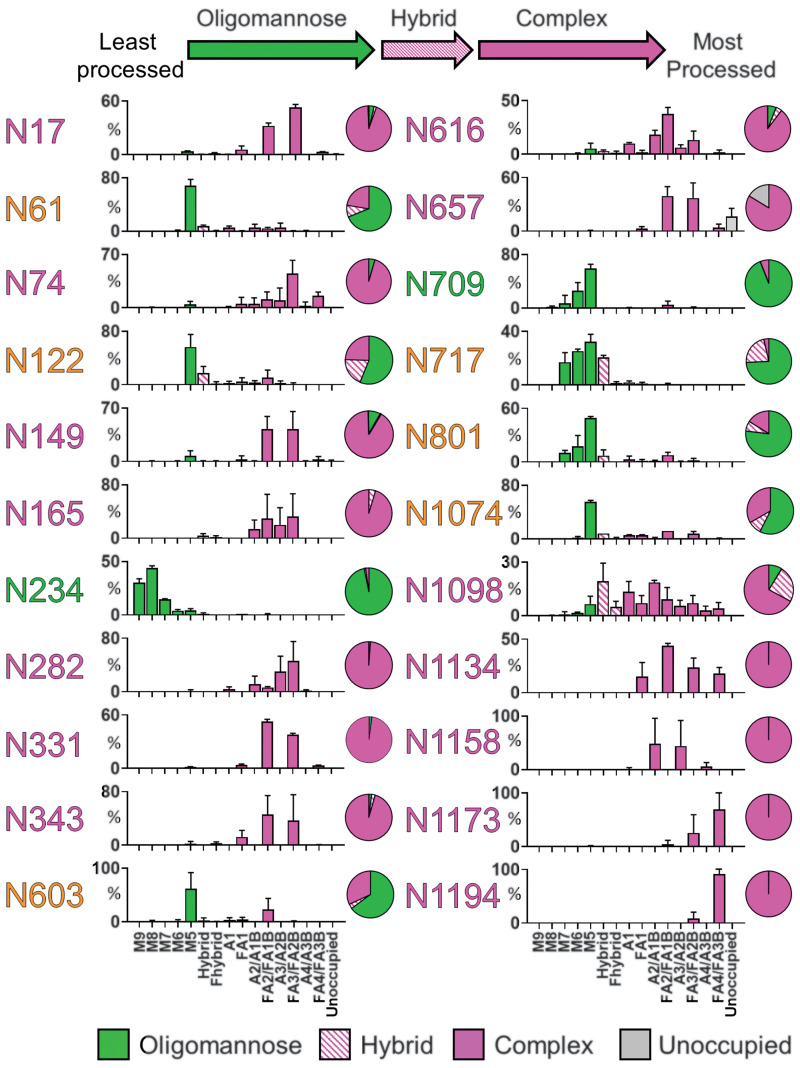
Site-specific N-linked glycosylation of the SARS-CoV-2 S glycoprotein. The schematic illustrates the color code for the principal glycan types that can arise along the maturation pathway from oligomannose- to hybrid- to complex-type glycans. The graphs summarize quantitative mass spectrometric analysis of the glycan population present at individual N-linked glycosylation sites simplified into categories of glycans. The oligomannose-type glycan series (M9 to M5; Man_9_GlcNAc_2_ to Man_5_GlcNAc_2_) is colored green, afucosylated and fucosylated hybrid-type glycans (hybrid and F hybrid) are dashed pink, and complex glycans are grouped according to the number of antennae and presence of core fucosylation (A1 to FA4) and are colored pink. Unoccupancy of an N-linked glycan site is represented in gray. The pie charts summarize the quantification of these glycans. Glycan sites are colored according to oligomannose-type glycan content, with the glycan sites labeled in green (80 to 100%), orange (30 to 79%), and pink (0 to 29%). An extended version of the site-specific analysis showing the heterogeneity within each category can be found in table S1 and fig. S2. The bar graphs represent the mean quantities of three biological replicates, with error bars representing the standard error of the mean.

To determine the site-specific glycosylation of SARS-CoV-2 S, we used trypsin, chymotrypsin, and α-lytic protease to generate three glycopeptide samples. These proteases were selected to generate glycopeptides that contain a single N-linked glycan sequon. The glycopeptides were analyzed by liquid chromatography–mass spectrometry, and the glycan compositions were determined for all 22 N-linked glycan sites (Fig. 2). To convey the main processing features at each site, the abundances of each glycan are summed into oligomannose-type, hybrid-type, and categories of complex-type glycosylation based on branching and fucosylation. The detailed, expanded graphs showing the diverse range of glycan compositions are presented in table S1 and fig. S2.

Two sites on SARS-CoV-2 S are principally oligomannose-type: N234 and N709. The predominant oligomannose-type glycan structure observed across the protein, with the exception of N234, is Man_5_GlcNAc_2_ (Man, mannose; GlcNAc, N-acetylglucosamine), which demonstrates that these sites are largely accessible to α-1,2-mannosidases but are poor substrates for GlcNAcT-I, which is the gateway enzyme in the formation of hybrid- and complex-type glycans in the Golgi apparatus. The stage at which processing is impeded is a signature related to the density and presentation of glycans on the viral spike. For example, the more densely glycosylated spikes of HIV-1 Env and Lassa virus (LASV) GPC exhibit numerous sites dominated by Man_9_GlcNAc_2_ (*21*–*24*).

A mixture of oligomannose- and complex-type glycans can be found at sites N61, N122, N603, N717, N801, and N1074 (Fig. 2). Of the 22 sites on the S protein, 8 contain substantial populations of oligomannose-type glycans, highlighting how the processing of the SARS-CoV-2 S glycans is divergent from host glycoproteins (*25*). The remaining 14 sites are dominated by processed, complex-type glycans.

Although unoccupied glycosylation sites were detected on SARS-CoV-2 S, when quantified they were revealed to form a very minor component of the total peptide pool (table S2). In HIV-1 immunogen research, the holes generated by unoccupied glycan sites have been shown to be immunogenic and potentially give rise to distracting epitopes (*26*). The high occupancy of N-linked glycan sequons of SARS-CoV-2 S indicates that recombinant immunogens will not require further optimization to enhance site occupancy.

**Fig. 3:**
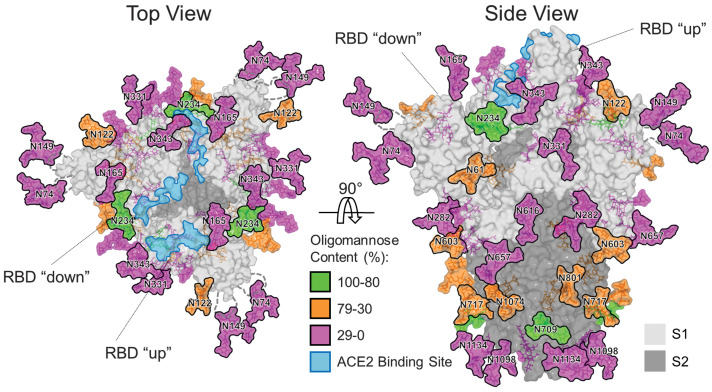
Structure-based mapping of SARS-CoV-2 S N-linked glycans. Representative glycans are modeled onto the prefusion structure of the trimeric SARS-CoV-2 S glycoprotein (PDB ID 6VSB) (*4*), with one RBD in the “up” conformation and the other two RBDs in the “down” conformation. The glycans are colored according to oligomannose content as defined by the key. ACE2 receptor binding sites are highlighted in light blue. The S1 and S2 subunits are rendered with translucent surface representation, colored light and dark gray, respectively. The flexible loops on which the N74 and N149 glycan sites reside are represented as gray dashed lines, with glycan sites on the loops mapped at their approximate regions.

Using the cryo-EM structure of the trimeric SARS-CoV-2 S protein [Protein Data Bank (PDB) ID 6VSB] (*4*), we mapped the glycosylation status of the coronavirus spike mimetic onto the experimentally determined three-dimensional (3D) structure (Fig. 3). This combined mass spectrometric and cryo-EM analysis reveals how the N-linked glycans occlude distinct regions across the surface of the SARS-CoV-2 spike.

Shielding of the receptor binding sites on the SARS-CoV-2 spike by proximal glycosylation sites (N165, N234, N343) can be observed, especially when the receptor binding domain is in the “down” conformation. The shielding of receptor binding sites by glycans is a common feature of viral glycoproteins, as observed on SARS-CoV-1 S (*10*, *13*), HIV-1 Env (*27*), influenza hemagglutinin (*28*, *29*), and LASV GPC (*24*). Given the functional constraints of receptor binding sites and the resulting low mutation rates of these residues, there is likely selective pressure to use N-linked glycans to camouflage one of the most conserved and potentially vulnerable areas of their respective glycoproteins (*30*, *31*).

We note the dispersion of oligomannose-type glycans across both the S1 and S2 subunits. This is in contrast to other viral glycoproteins; for example, the dense glycan clusters in several strains of HIV-1 Env induce oligomannose-type glycans that are recognized by antibodies (*32*, *33*). In SARS-CoV-2 S, the oligomannose-type structures are likely protected by the protein component, as exemplified by the N234 glycan, which is partially sandwiched between the N-terminal and receptor binding domains (Fig. 3).

We characterized the N-linked glycans on extended flexible loop structures (N74 and N149) and at the membrane-proximal C terminus (N1158, N1173, N1194) that were not resolved in the cryo-EM maps (*4*). These were determined to be complex-type glycans, consistent with steric accessibility of these residues.

Whereas the oligomannose-type glycan content (28%) (table S2) is above that observed on typical host glycoproteins, it is lower than other viral glycoproteins. For example, one of the most densely glycosylated viral spike proteins is HIV-1 Env, which exhibits ~60% oligomannose-type glycans (*21*, *34*). This suggests that the SARS-CoV-2 S protein is less densely glycosylated and that the glycans form less of a shield compared with other viral glycoproteins, including HIV-1 Env and LASV GPC, which may be beneficial for the elicitation of neutralizing antibodies.

Additionally, the processing of complex-type glycans is an important consideration in immunogen engineering, especially considering that epitopes of neutralizing antibodies against SARS-CoV-2 S can contain fucosylated glycans at N343 (*35*). Across the 22 N-linked glycosylation sites, 52% are fucosylated and 15% of the glycans contain at least one sialic acid residue (table S2 and fig. S3). Our analysis reveals that N343 is highly fucosylated with 98% of detected glycans bearing fucose residues. Glycan modifications can be heavily influenced by the cellular expression system used. We have previously demonstrated for HIV-1 Env glycosylation that the processing of complex-type glycans is driven by the producer cell but that the levels of oligomannose-type glycans were largely independent of the expression system and are much more closely related to the protein structure and glycan density (*36*).

**Fig. 4:**
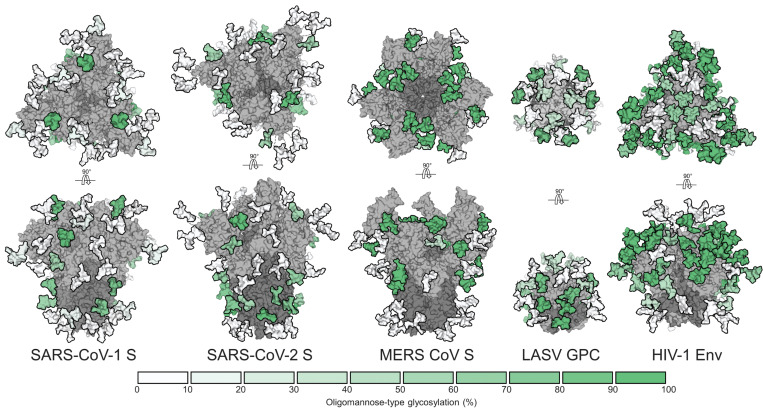
Underprocessing of viral glycan shields. From left to right, MERS-CoV S (*10*), SARS-CoV-1 S (*10*), SARS-CoV-2 S, LASV GPC (*24*), and HIV-1 Env (*8*, *21*). Site-specific N-linked glycan oligomannose quantifications are colored according to the key. All glycoproteins were expressed as soluble trimers in HEK 293F cells apart from LASV GPC, which was derived from virus-like particles from Madin-Darby canine kidney II cells.

Highly dense glycan shields, such as those observed on LASV GPC and HIV-1 Env, feature so-called mannose clusters (*22*, *24*) on the protein surface (Fig. 4). Whereas small mannose-type clusters have been characterized on the S1 subunit of Middle East respiratory syndrome (MERS)–CoV S (*10*), no such phenomenon has been observed for the SARS-CoV-1 or SARS-CoV-2 S proteins. The site-specific glycosylation analysis reported here suggests that the glycan shield of SARS-CoV-2 S is consistent with other coronaviruses and similarly exhibits numerous vulnerabilities throughout the glycan shield (*10*). Last, we detected trace levels of O-linked glycosylation at Thr^323^/Ser^325^ (T323/S325), with over 99% of these sites unmodified (fig. S4), suggesting that O-linked glycosylation of this region is minimal when the structure is native-like.

Our glycosylation analysis of SARS-CoV-2 offers a detailed benchmark of site-specific glycan signatures characteristic of a natively folded trimeric spike. As an increasing number of glycoprotein-based vaccine candidates are being developed, their detailed glycan analysis offers a route for comparing immunogen integrity and will also be important to monitor as manufacturing processes are scaled for clinical use. Glycan profiling will therefore also be an important measure of antigen quality in the manufacture of serological testing kits. Last, with the advent of nucleotide-based vaccines, it will be important to understand how those delivery mechanisms affect immunogen processing and presentation.

## Supplementary Materials

science.sciencemag.org/content/369/6501/330/suppl/DC1 Materials and Methods Figs. S1 to S4 Tables S1 and S2 References (*38*, *39*) MDAR Reproducibility Checklist Data S1

## Appendix

View/request a protocol for this paper from *Bio-protocol*.
